# Handheld Near-Infrared Spectroscopy Integrated with Pattern Recognition for Non-Destructive Discrimination of Rice Infected by an Aflatoxigenic Versus a Non-Aflatoxigenic *Aspergillus flavus* Isolate

**DOI:** 10.3390/foods15203762

**Published:** 2026-10-21

**Authors:** Zulipikaer Rouzi, Siyu Yao, Tong Yu, Ru Yan, Jin Wang, Xiyu He, Zuoyi Zhang, Luis E. Rodriguez-Saona

**Affiliations:** 1 Key Laboratory of Environmental Medicine and Engineering, Ministry of Education, and Department of Nutrition and Food Hygiene, School of Public Health, Southeast University, Nanjing 210009, China; 2 Nanjing Institute of Metrological Supervision and Testing, Nanjing 210009, China; 3 College of Food Science and Engineering, Inner Mongolia Agricultural University, Hohhot 010018, China; 4 Department of Food Science and Technology, The Ohio State University, Parker Food Science and Technology Building, 2015 Fyffe Road, Columbus, OH 43210, USA

**Keywords:** aflatoxigenic *Aspergillus flavus*, aflatoxin B_1_, handheld NIR sensor, pattern recognition analysis, early detection

## Abstract

Aflatoxin B_1_ (AFB_1_), a highly carcinogenic metabolite produced by aflatoxigenic *Aspergillus flavus*, poses a serious threat to rice safety and public health. This study aimed to establish a rapid, non-destructive method for discriminating between aflatoxigenic and non-aflatoxigenic *A. flavus* infection and for the exploratory classification of AFB_1_ contamination levels in rice by integrating a handheld near-infrared (NIR) sensor with pattern recognition analysis. A total of 154 post-harvest rice samples were inoculated with aflatoxigenic *A. flavus* or non-aflatoxigenic *A. flavus*, or left untreated as controls. Spectra were collected from each sample, and pattern recognition analysis (PCA, SIMCA, OPLS-DA) was used to discriminate between infection types and to categorize AFB_1_ content. The uHPLC-MS/MS reference method quantified AFB_1_ concentrations ranging from 2.70 to 23.59 ppb at early infection stages. PCA revealed distinct separation trends among the three sample groups. The SIMCA model achieved 100.00% accuracy, sensitivity, and specificity for discriminating among the three infection types in internal validation, and hold-out validation on an independent sample set demonstrated effective discrimination, yielding 96.67% overall accuracy. OPLS-DA produced comparable results, with accuracies of 95.90% in internal validation and 96.67% in hold-out validation. Notably, the SIMCA model was also applied in an exploratory manner to classify AFB_1_ content into low-AFB_1_ (<4.0 ppb) and high-AFB_1_ (≥4.0 ppb) groups; under Monte Carlo cross-validation and hold-out validation, this grading model yielded a mean accuracy of 63.10% and 87.50%, which demonstrated the potential feasibility of AFB_1_-level grading by handheld NIR spectroscopy. This approach shows potential for rapid laboratory-based screening in food supply chains to support timely risk assessment and intervention to protect food safety and public health.

## 1. Introduction

Aflatoxins are secondary metabolites predominantly produced by *Aspergillus flavus* (*A. flavus*) and *Aspergillus parasiticus*, and they readily contaminate agricultural products and feedstuffs (e.g., rice, corn, peanuts) during production, harvesting, storage, and food processing [1]. Their biosynthesis is a complex multistep biochemical process driven by diverse enzymatic reactions and metabolic intermediates within fungal cells [2]. Although both aflatoxigenic and non-aflatoxigenic *A. flavus* strains can infect agricultural products or feedstuffs, only aflatoxigenic strains possess the metabolic machinery required to synthesize aflatoxins [3]. According to the Food and Agriculture Organization of the United Nations (FAO), approximately one quarter of the world’s crops are contaminated with mycotoxins during growth or storage [4]. Aflatoxin production is strongly influenced by crop nutritional composition, water activity, and the relative humidity and temperature of the external environment [5].

Aflatoxins are derivatives of difuranocoumarins, of which 18 structural variants have been identified to date. Among these, aflatoxin B_1_, B_2_, G_1_, G_2_, M_1_, and M_2_ are the most prevalent aflatoxins in food commodities and feedstuffs [6]. These compounds exhibit mutagenic, teratogenic, and carcinogenic effects, imposing significant food safety and economic burdens [7]. Among all aflatoxins, aflatoxin B_1_ (AFB_1_) is the most toxic and is of the greatest regulatory concern. It is classified as a Group 1 human carcinogen by the International Agency for Research on Cancer (IARC) [8], and has been demonstrated to cause hepatotoxicity and neurotoxicity, as well as gastrointestinal, respiratory, and immune toxicity [9]. Moreover, aflatoxins are extremely thermally stable and cannot be eliminated or degraded by conventional cooking. Given their exceptional toxicity and prevalence, timely monitoring of AFB_1_ and *A. flavus* contamination in the food supply chain is critical. Rapid, non-destructive methods for identifying aflatoxigenic *A. flavus* and detecting AFB_1_ in foodstuffs are therefore imperative for ensuring food safety and protecting public health.

Rice is a staple food consumed by approximately half of the global population [10]. However, its high moisture-retention capacity makes it highly susceptible to *A. flavus* colonization and subsequent AFB_1_ accumulation [11,12]. Human exposure to AFB_1_ is a critical public health concern, particularly in developing countries, where inadequate storage conditions and limited regulatory oversight exacerbate the risk [13]. Early infection by *A. flavus* is metabolically dynamic, as fungal utilization of grain nutrients induces subtle biochemical changes that cannot be detected through visual inspection [14]. Conventional methodologies for AFB_1_ detection include high-performance liquid chromatography (HPLC), thin-layer chromatography (TLC), and enzyme-linked immunosorbent assay (ELISA). Although these methods are accurate and sensitive, they are time-consuming, require expensive instrumentation and laborious pretreatment with organic reagents, and are destructive, which precludes their use in large-scale, real-time, in situ screening [15]. Given these limitations, there is a growing demand for technologies that can rapidly and accurately identify aflatoxigenic *A. flavus* infection and AFB_1_ in rice non-destructively.

The combination of vibrational spectroscopy and pattern recognition analysis offers a feasible means to overcome the limitations of conventional detection methods. By linking spectral features with molecular functional groups, vibrational spectroscopy facilitates real-time acquisition of molecular fingerprint information [16]. Although mid-infrared (MIR) spectroscopy probes aflatoxin-related functional groups, it requires crushing and homogenization of the sample to achieve adequate detection accuracy [17]. Spontaneous Raman spectroscopy has been applied to the non-destructive detection of aflatoxins, but its detection limit (>100 ppb) is high owing to the weak inelastic scattering signal [18]. In contrast, near-infrared (NIR) spectroscopy has gained increasing attention for qualitative and quantitative analysis. Since *A. flavus* infection alters the physicochemical properties of rice, NIR may capture characteristic overtone and combination band changes associated with fungal growth and related metabolic processes, including those accompanying AFB_1_ production [19]. Most previous studies have compared rice inoculated with an aflatoxigenic strain against uninoculated controls, making it difficult to distinguish spectral variation potentially associated with aflatoxin-related metabolic changes from the broader effects of fungal infection [20,21,22]. Comparison of samples infected with aflatoxigenic and non-aflatoxigenic strains may provide additional information for exploring these different sources of spectral variation, although studies involving multiple strains of each type are needed to determine whether the observed patterns are generalizable beyond individual strains. Through preprocessing, dimensionality reduction, and feature extraction [23], pattern recognition analysis extracts characteristic spectral features of target analytes from complex matrices, supporting both classification and quantification tasks [24]. Miniaturized analytical systems, particularly handheld spectrometers, have been increasingly adopted in food safety monitoring due to their portability, rapid acquisition capability, and real-time analysis potential. When paired with mobile interfaces and embedded algorithms, handheld NIR devices can provide rapid contamination assessments directly at inspection sites, enabling immediate decision-making and intervention [25]. Their simplicity reduces operational barriers, supporting widespread adoption, and their non-destructive nature permits early detection and timely intervention.

The core objective of this study was to explore the feasibility of using NIR spectral sensing technology combined with pattern recognition analysis to qualitatively distinguish rice infected by the aflatoxigenic versus the non-aflatoxigenic *A. flavus* isolate investigated here, and to classify associated AFB_1_ content levels in an exploratory manner. This approach demonstrated the potential for non-destructive, rapid, and on-site monitoring of *A. flavus* infection and aflatoxin contamination in rice, and may offer a promising tool to enhance food safety protection.

## 2. Materials and Methods

**Figure 1:**
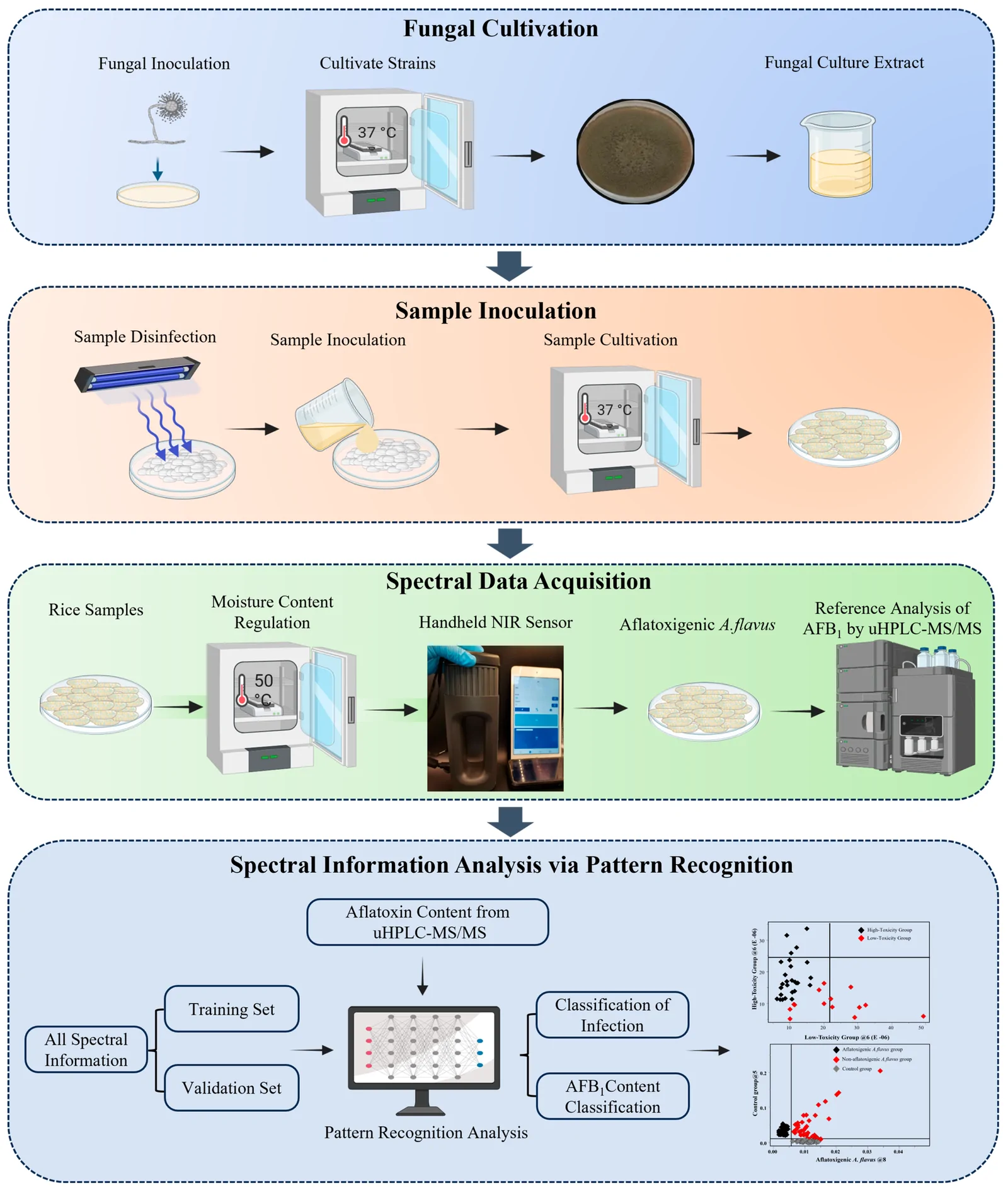
Overview of the experimental workflow for early-stage detection of *A. flavus* infection and AFB_1_ contamination in rice using handheld NIR spectroscopy coupled with chemometric analysis.

The overall experimental workflow is illustrated in Figure 1. This study comprised four stages, including fungal cultivation and spore suspension preparation, rice sample disinfection and inoculation, NIR spectral data acquisition with reference aflatoxin quantification by uHPLC-MS/MS, and chemometric pattern recognition analysis for infection status and AFB_1_ content classification.

### 2.1. Sample Collection and Inoculation

Rice samples were collected from a local grocery market and refrigerated at 4 °C until use in the analysis. Before inoculation, the rice was UV-sterilized in a biological safety cabinet (1500 Series A2; Thermo Scientific, Waltham, MA, USA) for 1 h to eliminate surface fungal contamination [26]. During this process, the rice samples were periodically turned over or agitated to ensure comprehensive surface sterilization. Then, rice samples (*n* = 154; approximately 3 g each) were randomly divided into three groups, which were inoculated with aflatoxigenic *A. flavus* (CGMCC 3.6304, China General Microbiological Culture Collection Center, Beijing, China, *n* = 49), inoculated with non-aflatoxigenic *A. flavus* (CICC 2476, China Center of Industrial Culture Collection, Beijing, China, *n* = 54), and left untreated as controls (*n* = 51), respectively. Plate culture checks confirmed no fungal growth in the controls throughout incubation. CICC 2476 (equivalent to ATCC 9643/DSM 1959) is documented by the corresponding culture collections as a non-aflatoxin-producing strain [27]; accordingly, uHPLC-MS/MS quantification of AFB_1_ was restricted to the samples infected by the aflatoxigenic isolate, which were used in the exploratory grading analysis.

The aflatoxigenic *A. flavus* and non-aflatoxigenic *A. flavus* isolates were cultured on V8 (Campbell Soup Company, Camden, NJ, USA) agar for 3 days at 30 °C in an incubator (DNP-9162Y; Jinghong, Shanghai, China) [17]. Conidia were harvested from the agar plates by washing three times with 15.0 mL of 0.1% (*v*/*v*) Tween 20 (Phygene, Fuzhou, China) solution. The conidial concentration was determined by microscopic enumeration using a hemocytometer. The suspension was then diluted with 0.1% Tween 20 solution to a final volume of 50 mL at 1 × 10^3^ CFU/mL. UV-sterilized rice samples were immersed in the aflatoxigenic inoculum, the non-aflatoxigenic inoculum, or a 0.1% Tween blank, respectively. Each sample was then transferred to a well of a six-well culture plate. The plate, with its lid slightly ajar, was placed in a plastic box lined with wet paper towels and incubated at 30 °C for 48 h to capture the early contamination stage; spectra were acquired immediately after incubation. Samples were then dried at 50 °C for 1 h to reduce free water and minimize baseline interference in the NIR spectra [28].

### 2.2. Spectral Data Acquisition

This experiment employed a handheld Fourier transform near-infrared (FT-NIR) sensor, the NeoSpectra-Scanner (Si-Ware Systems Inc., Cairo, Egypt) equipped with a MEMS-based, single-chip Michelson interferometer and offering a spectral resolution of 16 nm. Detection was performed with a single-element InGaAs photodetector. Before testing the samples, the instrument was calibrated against a 99% reflectance background. Each inoculated rice sample (~3 g) was placed into a sampling Petri dish and its spectral data were acquired over the spectral range of 1350–2500 nm (141 data points). The exposure time was set to 10 s. To maximize data reproducibility and signal-to-noise ratio (SNR), spectra of the samples were acquired in triplicate. The three replicate spectra of each sample were averaged to yield a single mean spectrum per sample. The spectrometer was recalibrated every hour to eliminate the effects of ambient light fluctuations or instrument drift on spectral stability. An iPad was wirelessly connected to the handheld NIR sensor via Bluetooth, and NeoSpectra Collect™ software (version 7.1.230328; Si-Ware Systems, Cairo, Egypt) was used to collect, store, and transmit the spectral data. Samples were snap-frozen in liquid nitrogen and stored at −20 °C pending uHPLC-MS/MS analysis.

### 2.3. uHPLC-MS/MS Aflatoxin Analysis

Aflatoxins were extracted from rice samples using the QuEChERS (Quick, Easy, Cheap, Effective, Rugged, and Safe) method and quantified by uHPLC-MS/MS [29]. Aflatoxin standard solutions (100 μg/mL in acetonitrile) were purchased from Tanmo Quality Testing Co., Ltd. (Changzhou, China). The reference material numbers for AFB_1_, AFB_2_, and AFG_1_ were 91661a, 91702a, and 91663a, respectively. Each rice sample was placed in a 50 mL centrifuge tube, and 40 μL of AFG_1_ (5 μg/mL) was added as the internal standard prior to extraction. The open-top centrifuge tubes containing the rice samples were placed under a biosafety hood for 20 min to evaporate the solvent. A volume of 15 mL of hexane (AR grade; Shanghai Lingfeng Chemical Reagent Co., Ltd., Shanghai, China) was added in batches to the centrifuge tube containing the sample. The rice–reagent mixture was homogenized using a handheld homogenizer. Subsequently, 2 mL of 0.1% formic acid (Macklin, Shanghai, China) and 13 mL of acetonitrile (HPLC grade; Aladdin, Shanghai, China) were added to the samples and mixed by rotation at 70 rpm for 20 min. A salt mixture containing 4 g of MgSO_4_, 1 g of NaCl, 1 g of trisodium citrate dihydrate (TSCD), and 0.5 g of disodium hydrogen citrate sesquihydrate (DHS) (Restek, Bellefonte, PA, USA) was added to each sample. The samples were rotated and mixed at 70 rpm for an additional 20 min (Rotating Mixer RML-80; JOANLAB, Hangzhou, Zhejiang, China). Finally, the samples were subjected to centrifugation at 4000 rpm for 20 min (Multifuge X1R Pro; Thermo Fisher Scientific, Waltham, MA, USA). Following centrifugation, the contents separated into three layers. The uppermost layer was the fat (hexane) layer, which was discarded. The acetonitrile solution in the middle layer was collected, and the white crystalline salt layer in the lower layer was discarded. The acetonitrile layer was subsequently transferred to a 2 mL dSPE (Restek, Bellefonte, PA, USA) tube containing 400 mg of primary secondary amine (PSA), 400 mg of octadecylsilane (C18), and 1200 mg of MgSO_4_. The dSPE tube was vortexed for 3 min and centrifuged at 4000 rpm for 10 min at 4 °C. The supernatant was filtered through a 0.22 μm PTFE filter (Jinteng, Tianjin, China) into an amber HPLC vial for uHPLC-MS/MS analysis.

Aflatoxin quantification was performed by uHPLC-MS/MS according to a previously reported method with minor modifications [30,31]. Standard curves were constructed using multi-level aflatoxin standard solutions containing known concentrations of the target AFB_1_, along with the internal standard AFG_1_. Analysis was performed on an AB SCIEX QTRAP 5500 LC-MS system equipped with an electrospray ionization (ESI) source operated in positive ion mode. Chromatographic separation was achieved on a Raptor ARC-18 column (150 × 3.0 mm, 2.7 μm; Restek, Bellefonte, PA, USA) with a guard column. Aflatoxins were monitored in MRM mode using the transitions *m*/*z* 313.10 > 241 and 313.10 > 285 for AFB_1_ and *m*/*z* 329.10 > 243 and 329.10 > 311 for AFG_1_ (IS). Mobile phase A was 0.1% formic acid in water, and mobile phase B was 0.1% formic acid in acetonitrile. The detailed gradient elution program is provided in Table S5. The flow rate and injection volume were set at 0.3 mL/min and 5 μL, respectively. Mixed standard working solutions of AFB_1_ were prepared by serial dilution. The dilution factor was 13. Calibration curves were constructed for AFB_1_ 0.000625–0.01 μg/mL, with correlation coefficients (R^2^) above 0.99. AFG_1_ was added to each sample and calibration standard prior to extraction at a fixed amount of 200 ng (40 μL of a 5 μg/mL solution), and quantification was performed using the AFB_1_/AFG_1_ peak-area ratio against the calibration curve. Spiking experiments on blank rice portions fortified with AFB_1_ at 10 and 20 μg/kg (4 and 8 μL of a 5 μg/mL AFB_1_ working solution) gave recoveries of 108.80% and 112.60%, respectively (mean 110.70%; RSD 2.40%), calculated by comparing the measured AFB_1_ concentrations with the corresponding fortification levels.

$$w=\frac{C\times V}{m}\times 1000$$

where *C* is the AFB_1_ concentration in the final extract (µg/mL) read from the calibration curve, *V* is the final extract volume (13 mL), and *m* is the individually weighed sample mass.

### 2.4. Pattern Recognition Analysis

To extract information associated with fungal infection and AFB_1_ contamination from the complex rice matrix, pattern recognition analysis was performed for model development and validation using Pirouette software (version 4.5; Infometrix Inc., Woodinville, WA, USA) and R (version 4.6.1; R Foundation for Statistical Computing, Vienna, Austria). Due to the high dimensionality, overlapping bands [25], and acquisition-induced noise of raw NIR spectra [32], preprocessing was applied to eliminate interferences, followed by pattern recognition for feature extraction and model construction [33]. Prior to model development, the dataset was randomly divided into a training set and an independent test set at a 4:1 ratio using stratified sampling to ensure proportional representation of each class in both subsets. The training set was used for model calibration and parameter optimization, and the independent test set was reserved exclusively for the final independent evaluation. Prior to preprocessing, spectral region selection was applied to reduce dimensionality and enhance the discriminative power of the models. Specifically, spectral regions at both ends, which were dominated by detector noise, and the strong water absorption region (1910–1950 nm, associated with O–H stretching and bending combinations) that could mask subtle biochemical signals related to fungal infection were excluded. The retained spectra were transformed by −log(X) conversion into pseudo-absorbance units, followed by standard normal variate (SNV) transformation and Savitzky–Golay smoothing with a 35-point window, and were mean-centered prior to model construction, reproducing the settings recorded in Pirouette to eliminate baseline drift, linear trend, and high-frequency noise for both classification models (SIMCA and OPLS-DA) [34]. 

Following preprocessing, principal component analysis (PCA) was first performed as an unsupervised exploratory tool. PCA decomposes the mean-centered spectral matrix X into mutually orthogonal components ordered by decreasing explained variance [35]. The resulting score matrix T describes the sample positions in the reduced component space, while the loading matrix P quantifies the contribution of each wavelength. To further establish predictive classification capabilities, supervised methods, namely soft independent modeling of class analogy (SIMCA, applied to both infection-type discrimination and AFB_1_ content grading) and orthogonal projections to latent structures–discriminant analysis (OPLS-DA, applied to infection-type discrimination), were then used to construct classification models [36]. SIMCA is a class-modeling technique in which an independent PCA model is developed for each predefined class [37]. Each class model defines an acceptance region jointly in the within-model (score) space, described by Hotelling’s $T_{i}^{2}=\sum_{k=1}^{A_{a}}\frac{t_{\mathrm{ik}}^{2}}{s_{k}^{2}}$, and in the orthogonal residual space, described by $Q_{i}=\underset{j}{\sum }e_{\mathrm{ij}}^{2}$, with both statistics evaluated against their 95.00% critical limits (the threshold lines in the class distance plot); a sample is assigned to a class only when it satisfies both criteria, and, because the class models are independent, a sample may belong to several classes or to none (outlier), which constitutes the “soft” classification rule. OPLS-DA was included as a complementary regression-based approach. While SIMCA models each class independently, OPLS-DA combines partial least squares (PLS) regression with discriminant analysis by maximizing the covariance between the spectral matrix X and the class response Y [38], while filtering out the orthogonal variation (systematic noise) unrelated to the class membership [39]. The number of components retained in each class model was determined by evaluating the explained variance and the cross-validation performance across increasing component numbers. A configuration was retained when further increases in the number of components produced no substantial additional improvement in explained variance or cross-validation performance, so as to balance model complexity against the risk of overfitting.

To evaluate model performance and prevent overfitting, leave-one-out cross-validation (LOOCV) was employed on the training set as an internal validation strategy. In this iterative procedure, each sample is sequentially left out as a test set while the model is trained on the remaining n − 1 samples, providing a nearly unbiased estimate of the prediction error and guiding the selection of model complexity [40]. Subsequently, hold-out validation was performed by applying each model calibrated on the training set to the reserved independent test set in a single run, thereby providing an estimate of predictive performance on samples excluded from calibration and parameter optimization. Beyond parameter optimization, Monte Carlo cross-validation (MCCV) was additionally performed on the full dataset to examine whether the performance of both the infection classification models and the AFB_1_ grading model depended on the particular data partition [41]. For each model, 200 random stratified 4:1 partitions of the full dataset were performed, so that the consistency of the reported performance metrics across partitions, rather than artifacts of a specific data split, could be assessed [42]. To test whether the observed discrimination could arise from chance correlation, a Y-scramblingtest was additionally performed with 200 iterations for all models. By randomly permuting the class labels relative to the spectral data, this test indicated whether the observed performance exceeded the chance level established under label permutation [38].

To provide a comprehensive statistical evaluation, model-specific metrics were further calculated. For the SIMCA models, class separation was quantified by the interclass distance (ICD) between classes a and b: $\mathrm{ICD}\left(a,b\right)=\sqrt{\frac{s_{b\to a}^{2}}{s_{a}^{2}}+\frac{s_{a\to b}^{2}}{s_{b}^{2}}-2}$, where $s_{a\to b}^{2}$ is the residual variance obtained when the samples of class a are fitted to the model of class b, and $s_{a}^{2}$ is the residual variance of class a fitted to its own model; ICD values greater than 3 indicate well-separated classes [16]. In addition, the discriminating power of each wavelength was used to identify the variables contributing most to class separation. For the OPLS-DA models, the root mean square error of cross-validation (RMSECV), estimated by LOOCV on the training set, was used to guide the selection of the number of latent variables. The stability of both models under different data partitions was assessed by MCCV, with the performance metrics across the 200 partitions reported as mean ± standard deviation. The statistical significance of the models was verified by the Y-scrambling permutation test, with the *p*-value calculated as (k + 1)/(N + 1), where k is the number of permuted models achieving a performance equal to or better than that of the true model and N is the total number of permutations (*N* = 200). Finally, classification performance was quantified by accuracy, sensitivity, and specificity. Accuracy was defined as the proportion of all samples correctly assigned to their true class. Sensitivity and specificity were calculated on a one-vs-rest basis and pooled across all classes, expressed as ΣTP/ (ΣTP + ΣFN) and ΣTN/(ΣTN + ΣFP), respectively, where TP and FP denote the numbers of samples correctly and incorrectly assigned to a given class, and FN and TN denote the numbers of class members and non-members not assigned to that class [43].

## 3. Results and Discussion

### 3.1. Contamination Dynamics of A. flavus in Rice and AFB_1_ Quantification by uHPLC-MS/MS

This study focused on acquiring NIR spectra during the early, low-contamination stage of *A. flavus* infection. To capture genuine early-stage infection without interference from advanced mycelial proliferation, the inoculum concentration and incubation time were optimized (Supplementary Figure S3). Under these conditions, no visible mycelial growth was observed at 24 h post-inoculation, while light green mycelium appeared by 48 h post-inoculation, consistent with the study’s aim of capturing early-stage infection. Visual inspection cannot reliably distinguish rice infected by aflatoxigenic strains from that infected by non-aflatoxigenic strains, making spectral analysis essential. This approach is consistent with the artificial inoculation strategies employed by Wang et al. (2015) and Guo et al. (2023) on crops to capture subtle spectral variations associated with early fungal proliferation and initial toxin accumulation, which further supports that the inoculum concentration and observation period used here were appropriate for capturing early-stage infection [44,45]. After inoculation, rice samples infected with aflatoxigenic *A. flavus* were analyzed to determine their AFB_1_ content, which ranged from 2.70 to 23.59 ppb (Table 1). Overall, the toxin levels remained relatively low during the early infection period, although measurable variation was observed across samples. Based on these measurements, the aflatoxigenic *A. flavus*-infected samples were stratified into two groups, with those containing less than 4.0 ppb designated as the low-AFB_1_ group and those at or above 4.0 ppb classified as the high-AFB_1_ group. The corresponding NIR spectral data were subsequently analyzed using SIMCA to assess whether the model could classify samples according to spectral characteristics associated with their AFB_1_ content. The AFB_1_ levels in this study remained low, reflecting the reduced inoculum concentration and shorter incubation period used here.

### 3.2. Characterization of Sample Spectral Information

**Figure 2:**
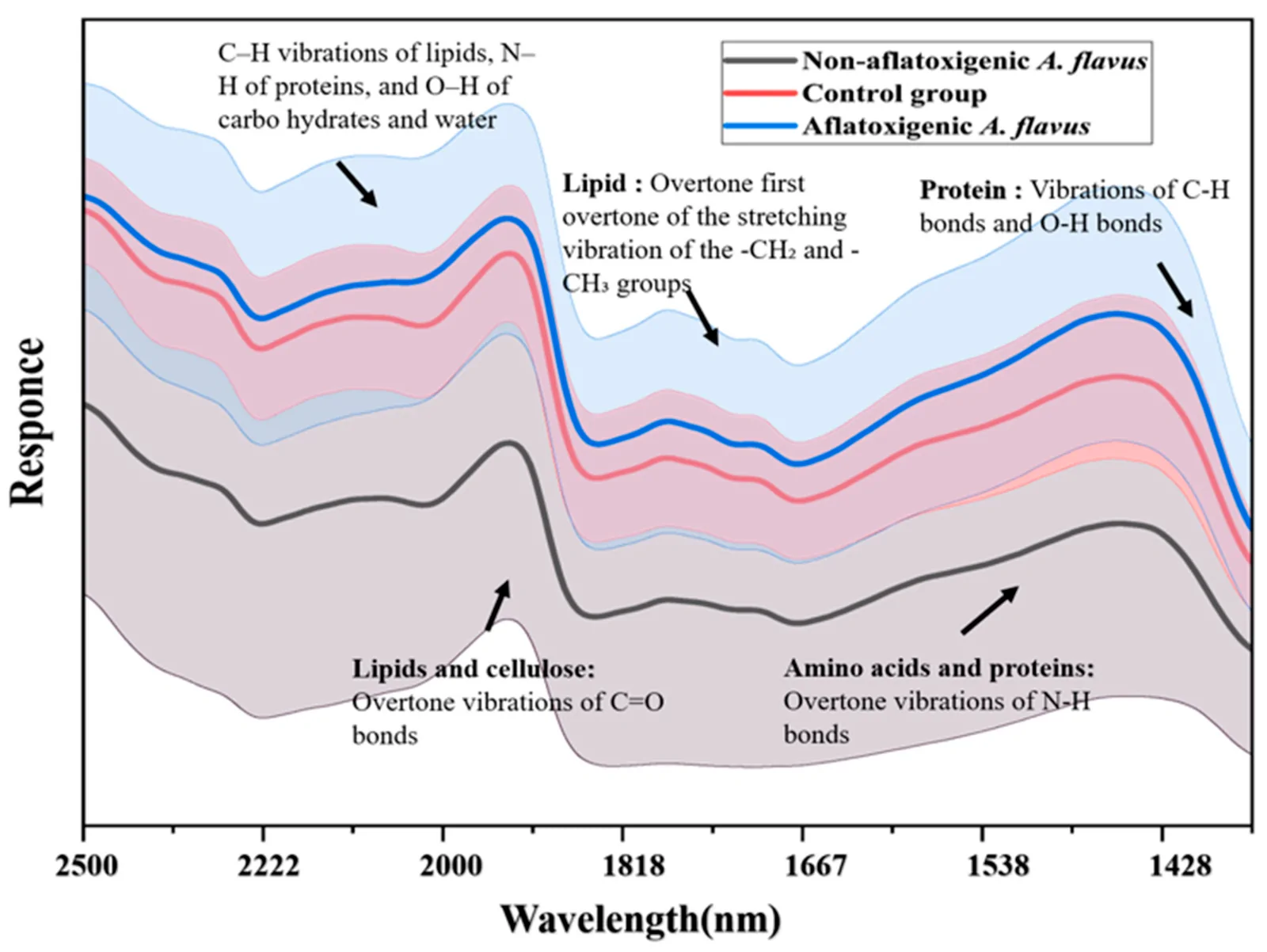
Average spectra with corresponding standard deviation ranges for the aflatoxigenic *A. flavus* group, non-aflatoxigenic *A. flavus* group, and the control group.

As shown in Figure 2, the solid lines show the mean spectrum of each group and the shaded regions indicate the corresponding standard deviations, highlighting both the overall spectral profiles and intra-group variability. The three groups separate most clearly in the N–H-related region (1428–1518 nm; protein) and the C–H-associated region (1647–2000 nm; lipids and cellulose). The broader shaded regions indicate greater spectral variability; likely reflecting group differences associated with infection status and isolate identity. Baseline offsets likely arise from light scattering caused by infection-related changes in grain surface and internal structure. The 1410–1450 nm band arises from C–H and O–H vibrations in proteins, and the 1510–1540 nm band from N–H overtone vibrations in amino acids and proteins. The 1700–1800 nm band corresponds to the first overtone of –CH_2_ and –CH_3_ stretching and characterizes lipids. Absorption at 1910–1950 nm is associated with the first overtone of O–H stretching and O–H bending–stretching combinations. The 2010–2200 nm region involves N–H and C=O stretching and combination bands. The 2250–2400 nm region primarily reflects C–H combination vibrations [46,47,48,49]. The spectral variability is consistent with metabolic dynamics during the early fungal colonization process, in which the consumption of rice proteins and lipids may induce measurable alterations in NIR spectral signatures; because fungal biomass was not quantified, the effect of differences in fungal growth extent cannot be separated from isolate-specific metabolic effects. Furthermore, grains in the field are susceptible to infection by multiple fungal strains and to interference from other physicochemical factors. These confounding factors jointly affect grain samples and would complicate the spectral analysis of field-collected samples [32].

### 3.3. PCA-Based Pattern Recognition Analysis of NIR Spectra from A. flavus-Infected and Control Rice Samples

**Figure 3:**
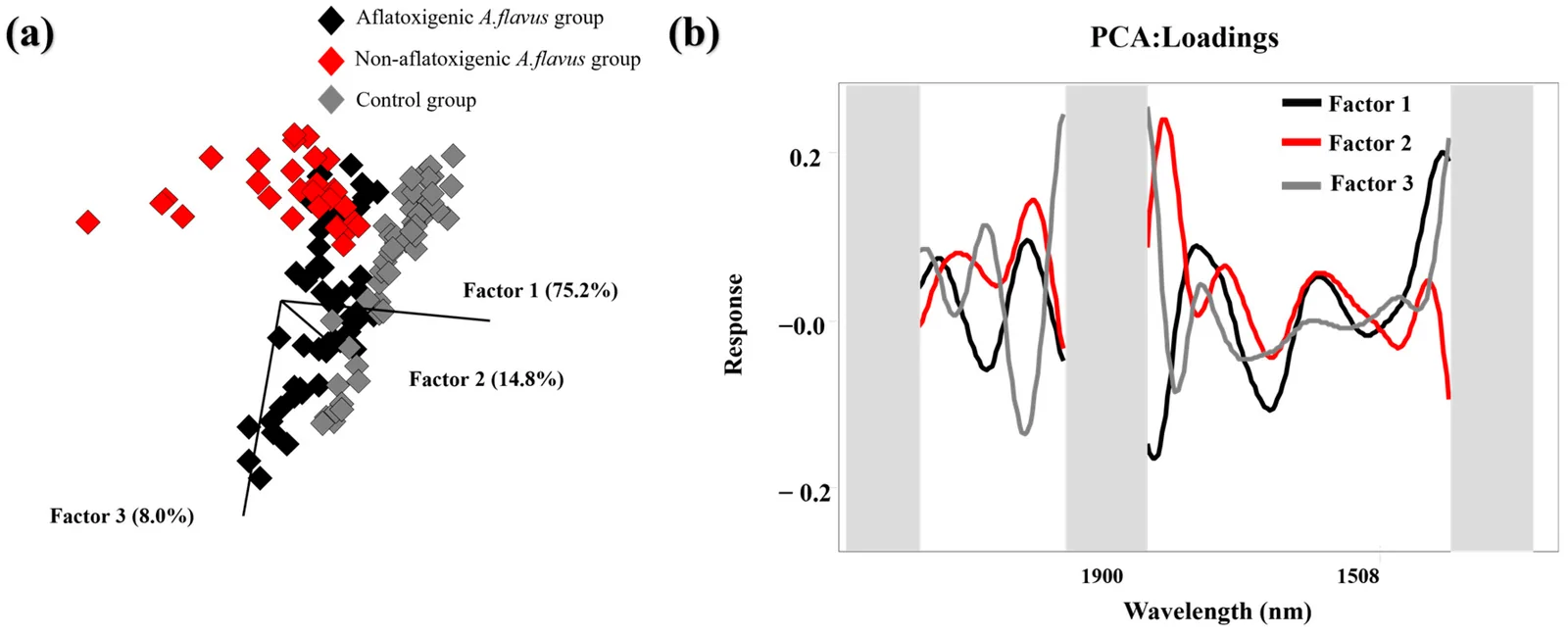
(**a**) PCA score plot constructed using the first three principal components; and (**b**) the corresponding loading plot.

PCA was first applied, revealing separation trends among the aflatoxigenic *A. flavus*-infected, non-aflatoxigenic *A. flavus*-infected, and control groups in the three-dimensional score space (Figure 3a). The spectral differences between the two infected groups may reflect an underlying biochemical distinction, possibly involving divergent toxin metabolism, isolate-specific growth, or other physiological factors, and thus suggest the feasibility of isolate-level discrimination. The control group formed a separate cluster, indicating that fungal contamination can produce detectable spectral signatures. The first three principal components accounted for 98.00% of the cumulative variance (Factor 1: 75.20%, Factor 2: 14.80%, Factor 3: 8.00%). Factor 1 was identified as the dominant discriminating dimension. The PCA loading plots illustrate the strength of the correlation between each principal component and the original variables (wavelengths) (Figure 3b). Specifically, the larger the absolute value of a loading, the greater the contribution of the corresponding wavelength to that principal component; the sign (positive/negative) of the loading indicates the direction of the correlation between the original variable and the principal component. Notably, prominent peaks at 2200 nm (N–H and C=O) and 1500 nm (amino acids and proteins) were observed. These peaks are likely attributed to the vibrations of specific chemical bonds or functional groups, serving as the most prominent features for explaining the variance. These protein- and lipid-related regions may therefore reflect growth-associated consumption of rice proteins and lipids by *A. flavus*. Changes in signal intensity in these regions account for most of the spectral variability [50].

### 3.4. SIMCA-Based Pattern Recognition of NIR Spectra from A. flavus-Infected and Control Rice Samples

Although PCA enables spectral data dimensionality reduction and extraction of characteristic features from the aflatoxigenic *A. flavus*, non-aflatoxigenic *A. flavus*, and control groups, it cannot directly classify unknown samples during predictions [51]. SIMCA, by contrast, assigns new samples to predefined classes and is therefore suited to tasks such as discriminating rice infected by the investigated aflatoxigenic versus non-aflatoxigenic isolate and distinguishing infected from uninfected controls. SIMCA was therefore used to model the spectra in three classes: aflatoxigenic-inoculated, non-aflatoxigenic-inoculated, and control.

**Figure 4:**
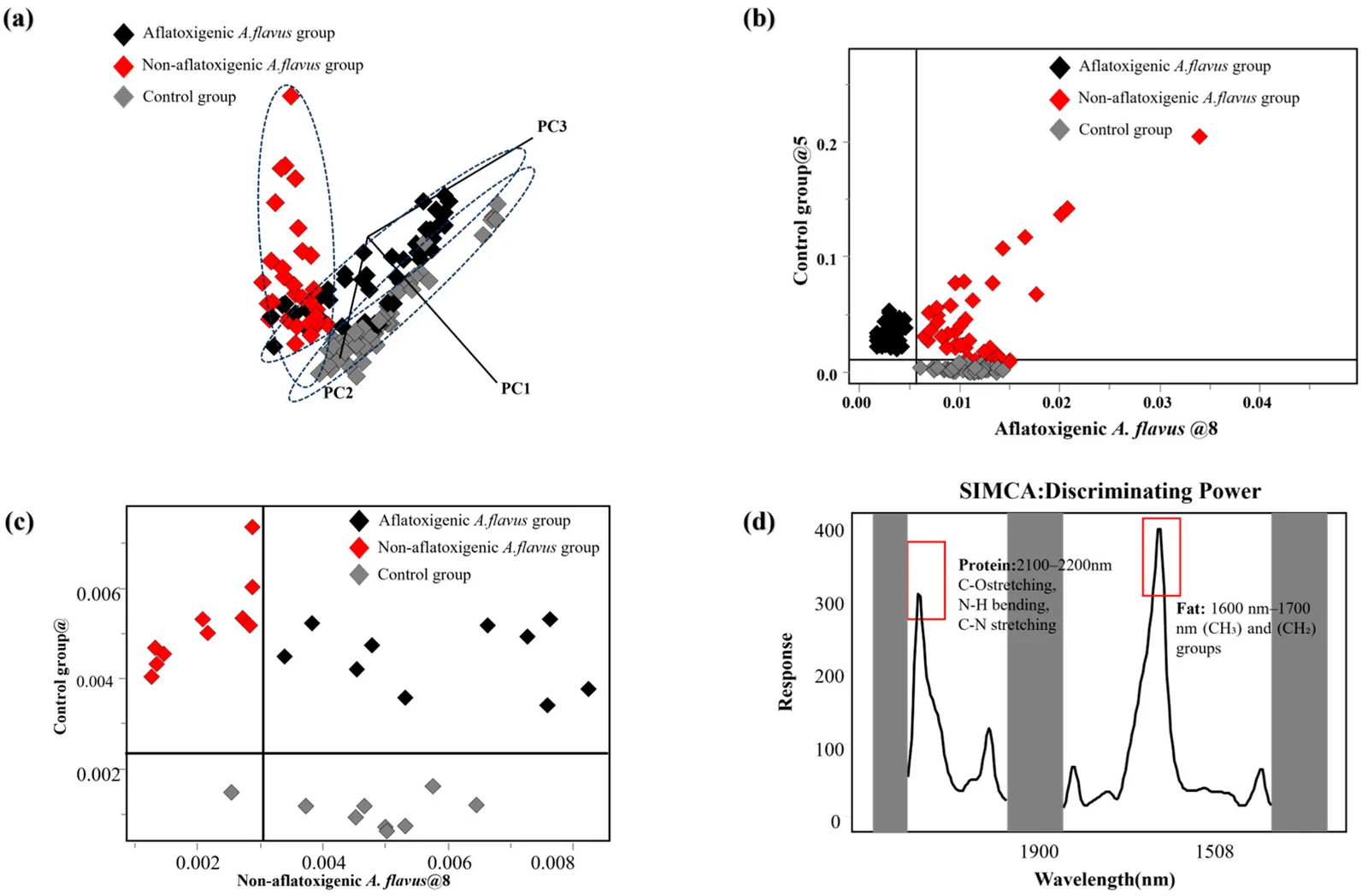
SIMCA classification results displaying: (**a**) a 3D projection plot with distinct cluster separation among the three groups; (**b**) a class distance plot differentiating the sample groups; (**c**) a corresponding plot for hold-out validation; and (**d**) a discriminating power plot identifying key functional groups from the NIR spectra. The symbols @8 and @5 represent the optimal number of principal components used to model the aflatoxigenic and non-aflatoxigenic *A. flavus* groups, respectively, selected to maximize variance explanation while preventing overfitting.

The SIMCA Class Projections plot shows compact, well-separated clusters for all groups, with sample points distributed within a narrow dispersion range. The tight clustering of biological replicates indicates low intra-group variability (Figure 4a). The SIMCA model demonstrated the capability to distinguish among different groups. Although the two inoculated groups involve the same species and share the same rice matrix, their classification suggests the feasibility of this approach under the controlled single-isolate conditions. In the PC1–PC3 projection space, the three groups occupied clearly separated regions. The well-defined boundaries among the control, aflatoxigenic *A. flavus*-inoculated, and non-aflatoxigenic *A. flavus*-inoculated groups indicate that the inoculation treatments induced measurable biochemical changes. The SIMCA residuals for each sample are shown in the class distance plot (Figure 4b). The two threshold lines mark the critical values for the aflatoxigenic *A. flavus* and control classes. These lines divide the diagram into four quadrants. Samples in the northwest quadrant belong exclusively to the aflatoxigenic *A. flavus* group, while those in the southeast quadrant belong solely to the control group. Samples in the southwest quadrant may belong to either category, while those in the northeast quadrant do not belong to either the aflatoxigenic *A. flavus* group or the control group. Thus, in the present dataset, all samples were uniquely assigned to a single class. Class separation was quantified by interclass distance (ICD), for which values above 3 are generally considered to indicate excellent separation. The ICDs among the three groups were 4.04, 5.23, and 9.98 (Table 2) [52]. The model was further evaluated by LOOCV, achieving 100.00% accuracy, sensitivity, and specificity across the 124 training samples. Hold-out validation on the reserved test split then yielded an overall accuracy of 96.67%, sensitivity of 96.67%, and specificity of 100.00% (Table 3). To examine whether this single-split estimate depended on the particular data partition, MCCV was performed with 200 random stratified 4:1 partitions of the full dataset. Across these 200 iterations, the SIMCA model achieved an average accuracy of 94.14 ± 4.53% with a 95.00% confidence interval of 84.40–100.00% (Supplementary Table S3), and the hold-out accuracy lies within this interval, indicating that the reserved split is not an outlier of the partition distribution. In addition, a Y-scrambling test (Supplementary Table S7 and Figure S4) yielded a permuted accuracy of 32.79 ± 4.18% (mean ± SD), and the observed accuracy exceeded all 200 permuted values (*p* < 0.005), indicating that the discrimination is unlikely to arise from chance association between the spectra and the class labels. Taken together, these results indicate that the SIMCA model achieved acceptable performance in discriminating the three infection types under the controlled single-strain and single-batch conditions, suggesting that this approach has potential for rapid identification of *A. flavus* infection types.

The class distance plot for the hold-out validation set confirmed accurate classification of the independent samples (Figure 4c). Collectively, these results suggest that the model possesses acceptable recognition capability across the investigated categories and exhibits a consistent ability to mitigate interfering signals, thereby supporting its potential applicability for multi-class classification tasks under the controlled single-strain and single-batch conditions. SIMCA’s discriminating power facilitates the identification of the most critical inter-class variables, with its corresponding plot displaying the contribution values of each band. As illustrated in Figure 4d, the 1600–1700 nm and 2100–2200 nm bands exhibited strong contributions, corresponding to the (CH_3_) and (CH_2_) lipid groups, respectively, and vibrations of N–H and C=O bonds, respectively [47]. This observation aligns with Zhao et al. [53], who reported that lipid- and protein-related constituents in rice facilitate *A. flavus* growth and metabolism, supporting our finding.

### 3.5. OPLS-DA-Based Pattern Recognition of NIR Spectra from A. flavus-Infected and Control Rice Samples

Because NIR spectra are highly dimensional and collinear, OPLS-DA uses orthogonal filtering to remove variation unrelated to class membership, thereby improving class discrimination. OPLS-DA combines partial least squares regression with discriminant analysis to model the relationship between the spectral matrix X and the class response Y [54]. In this study, the response matrix (Y, sample class) corresponds to a predefined qualitative variable, while the descriptive matrix (X) comprises the NIR spectral data acquired in the aforementioned experimental procedures. The combined use of SIMCA and OPLS-DA therefore reduces the risk of false positives/negatives inherent in single-model analyses.

**Figure 5:**
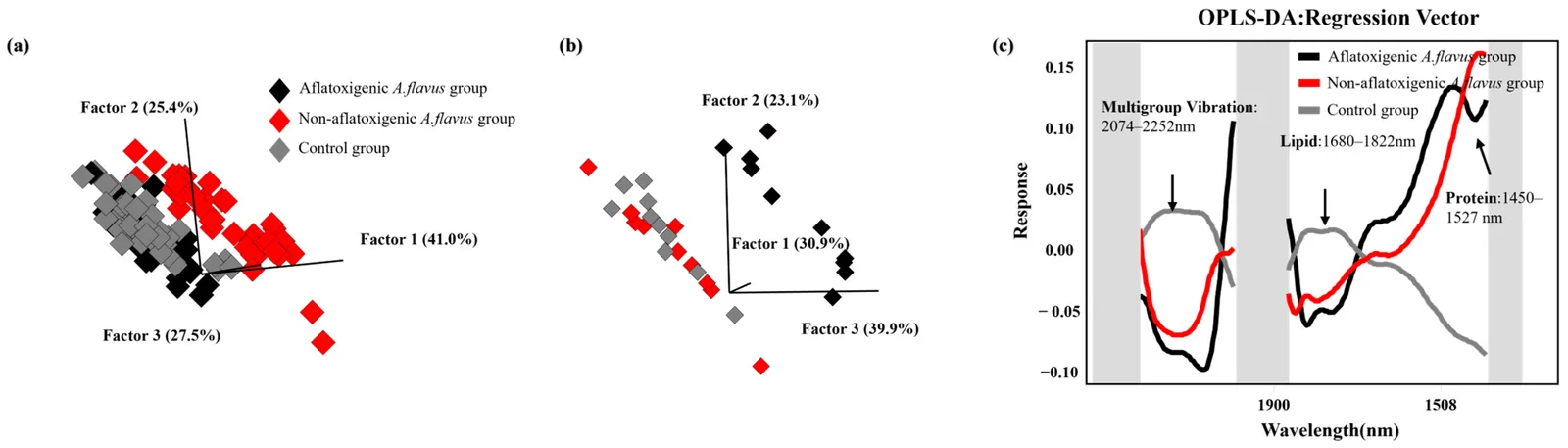
(**a**) OPLS-DA score plot; (**b**) hold-out validation score plot and (**c**) its corresponding regression vector derived from NIR spectral data of the aflatoxigenic *A. flavus* group, non-aflatoxigenic *A. flavus* group and the control group.

As shown in Figure 5a, OPLS-DA produced well-segregated spatial clusters, indicating clear inter-group discrimination and suggesting that the three groups may exhibit distinct biochemical alterations (e.g., metabolite profiles). In addition, compact clustering within each group reflects low intra-group variability, suggesting shared metabolic characteristics among samples within the group. As shown in Table 3, LOOCV results from OPLS-DA analysis demonstrated an accuracy of 95.90%, sensitivity of 95.90%, and specificity of 99.18%, for differentiating aflatoxigenic *A. flavus*, non-aflatoxigenic *A. flavus*, and the control group. Hold-out validation was then performed on the reserved test split, and the model achieved an overall accuracy of 96.67%, sensitivity of 96.67%, and specificity of 98.33%. To examine whether the single-split hold-out estimate depended on the particular data partition, MCCV was performed with 200 random stratified 4:1 partitions of the full dataset. Across these 200 iterations, the OPLS-DA model maintained highly stable performance, achieving an average accuracy of 95.64 ± 3.52% with a 95.00% confidence interval of 87.50–100.0% (Supplementary Table S3). As illustrated by the two models in the present study, the relative performance of LOOCV and MCCV was not consistent across modeling approaches. LOOCV yielded a higher accuracy than MCCV for SIMCA (100% vs. 94.14 ± 4.53%), whereas the opposite pattern was observed for OPLS-DA (95.90% vs. 95.64 ± 3.52%). LOOCV and MCCV are distinct resampling procedures with different statistical properties, and their resulting performance estimates are influenced not only by the size of the training set but also by the model and the composition of the validation subsets [41,55]. In addition, a Y-scrambling permutation test (200 permutations; Supplementary Table S4 and Figure S2) produced a permuted accuracy of 32.69 ± 5.42% (mean ± SD), with *p* < 0.005, supporting that the model’s performance arises from genuine spectral–class relationships rather than chance correlations. Model performance was further assessed using cumulative explained variance and root mean square error of validation (RMSECV). For the aflatoxigenic and non-aflatoxigenic *A. flavus* groups, the first three factors captured over 93.00% of the variance (93.85% and 93.78%, respectively). For the control group, four factors were required to reach 97.09% cumulative variance (78.58% for the first three). Thus, the essential variation in all three groups was captured within the first three to four factors, with little gain thereafter. RMSECV decreased through factor five but increased from factor six onward, indicating that adding more factors leads to overfitting (Table 4). Overall, OPLS-DA achieved acceptable performance in discriminating the three infection types and showed potential for this task, while confirmation on independent multi-batch data remains warranted.

In the hold-out validation score plot (Figure 5b), samples of the same group clustered tightly while different groups remained well separated, consistent with the discrimination. The OPLS-DA regression factor plot shows how the spectral responses differ between groups at various wavelengths, helping to identify the regions of the spectrum that distinguish each group (Figure 5c). The aflatoxigenic *A. flavus* group (black curve) and non-aflatoxigenic *A. flavus* group (red curve) exhibited markedly reduced responses in the protein-related wavelength region (1500–1600 nm, amino acids/proteins) and the combination-band region (2100–2200 nm). In contrast, the control group (grey curve) showed a relatively higher response at these positions. This indicated that the protein-associated wavelength range (1500–1600 nm) and multigroup vibration region (2100–2200 nm) serve as key indicators for distinguishing *A. flavus*-infected rice samples from uninfected controls. This may reflect the consumption of proteins and amino acids in the rice matrix by fungi at the early infection stage. As a hypothesis, the additional metabolic burden associated with aflatoxin B_1_ biosynthesis may alter the consumption pattern of these nutrients in the aflatoxigenic isolate relative to the non-aflatoxigenic isolate, and the observed band differences may therefore reflect such toxin-related metabolic differences. These results are consistent with the aforementioned PCA and SIMCA model outcomes, further supporting the stability of the constructed models in the present study. 

### 3.6. Pattern Recognition Analysis for Exploratory Grading of AFB_1_ Content in Aflatoxigenic A. flavus-Infected Rice Using PCA and SIMCA

**Figure 6:**
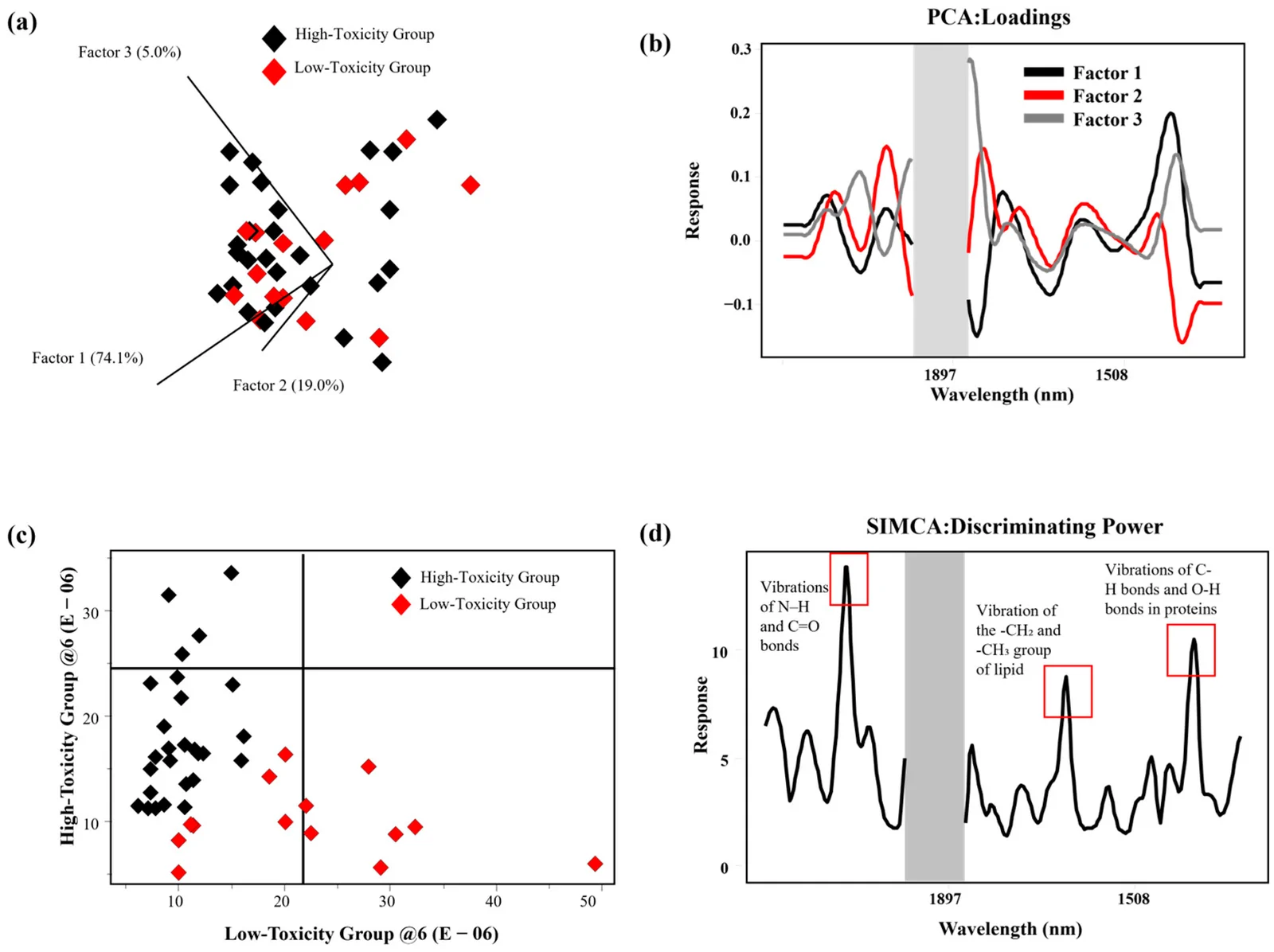
(**a**) PCA score plot; (**b**) PCA loading plot; (**c**) SIMCA class distance plot for low-AFB_1_ and high-AFB_1_ sample groups; and (**d**) discriminating power plot generated from the NIR spectral data of the aforementioned two groups (low-AFB_1_ and high-AFB_1_ groups). The symbols @6 represent the optimal number of principal components used to model the low- and high-AFB_1_ groups selected to maximize variance explanation while preventing overfitting.

In this study, uHPLC-MS/MS was employed as the gold standard for quantifying AFB_1_ in aflatoxigenic *A. flavus*-infected samples, and pattern recognition analysis was used to classify samples according to AFB_1_ content. Under Regulation (EU) 2023/915, cereals placed on the market for the final consumer, including the retail rice used here, are subject to maximum levels of 2 μg/kg for AFB_1_ and 4 μg/kg for total aflatoxins; the higher 5 μg/kg AFB_1_ limit applies only to rice intended for further sorting or physical treatment. Because all measured AFB_1_ concentrations in this study (2.70–23.59 ppb) exceeded the 2 μg/kg consumer limit, a legally defined cutoff would have left no compliant class for model training. A threshold of 4.0 ppb was therefore adopted as an exploratory, data-driven cutoff for early-stage contamination, informed by the experimental data distribution and practical screening needs. Samples with AFB_1_ ≥ 4.0 ppb were assigned to the high-AFB_1_ group (*n* = 17) and those below 4.0 ppb to the low-AFB_1_ group (*n* = 32). Of these 49 samples, leaving 42 samples (27 low-AFB_1_, 2.70–3.94 ppb; and 15 high-AFB_1_, 4.07–23.59 ppb). For model development, these 42 samples were split at a 4:1 ratio into a 34-sample training set (22 low-AFB_1_ and 12 high-AFB_1_) and an eight-sample independent hold-out set (5 low-AFB_1_ and 3 high-AFB_1_). During the initial data-screening stage, seven samples (5 with mean concentrations below 4.0 ppb and two with mean concentrations at or above 4.0 ppb) were not retained for the binary grading analysis because their replicate uHPLC-MS/MS measurements straddled the exploratory 4.0 ppb cutoff. PCA was first employed to explore the intrinsic distribution of the dataset. In the three-dimensional score space, the PCA score plot showed slight overlap between the two groups (especially in the middle region), with no distinctly separated clusters formed. This suggests that the feature dimensions represented by the first three principal components do not fully differentiate the high- and low-AFB_1_ samples (Figure 6a). The first three principal components collectively explain 98.10% of the variance (Factor 1: 74.10%; Factor 2: 19.00%; Factor 3: 5.00%). Through PCA loading analysis, the core contributing wavelengths of the original spectra in the first three principal components were identified at around 1400 nm (protein) (Figure 6b). In the SIMCA class distance plot (Figure 6c), the vertical and horizontal lines represent the 95.00% confidence limits of the low-AFB_1_ and high-AFB_1_ classes, respectively; samples plotting in the left-upper or lower-right quadrant are accepted by only one class, whereas samples located within both confidence limits, i.e., in the lower-left (southwest) quadrant, are accepted by both class models. Several samples were observed in this overlapping region, reflecting the close similarity of their spectral profiles to those of the opposite class at such low contamination levels.

Supervised classification was then applied. Given the sample size (*n* = 42), SIMCA was preferred over OPLS-DA to minimize the risk of overfitting. In the SIMCA class distance plot, several samples fell into the southwest quadrant, indicating membership in both the high- and low-AFB_1_ classes. Nevertheless, the model achieved 100.00% accuracy, sensitivity, and specificity in internal validation (Table 5). The hold-out validation yielded an overall accuracy of 87.50%, with a sensitivity of 100.00% (5/5) for the low-AFB_1_ group and a specificity of 66.67% (2/3) for the high-AFB_1_ group. The model was further assessed with MCCV and a Y-scrambling test (Supplementary Table S6). The four-component model was used exclusively for the MCCV and the Y-scrambling test. Because these two tests rebuild the model from scratch on a smaller training subset in each of the 200 iterations, the supportable model complexity is lower than that of the final model; accordingly, this configuration was selected because it yielded the highest mean balanced accuracy in the component scan (Supplementary Table S6), and increasing the number of components beyond four did not result in consistent improvement. In addition, the Y-scrambling test yielded an empirical *p* value of 0.1540. Thus, under the current dataset and validation setting, the permutation analysis did not provide statistically significant evidence that the observed discrimination was consistently distinguishable. These findings suggest that the model performance may be sensitive to sampling variability and potential chance associations, and that its reproducibility and generalizability require further validation. In each MCCV iteration, the 42-sample dataset was partitioned at a 4:1 ratio, leaving approximately eight samples in the test subset, typically including only two to three high-AFB_1_ samples. As a result, partition-to-partition variability was large, and a single misclassification could shift the balanced accuracy by 15–25 percentage points. In addition, the relatively low AFB_1_ concentrations (2.70–23.59 ppb) and subtle spectral differences between the low- and high-AFB_1_ groups may further increase the difficulty of obtaining stable discrimination. Therefore, rather than interpreting the internal and hold-out results in isolation, we now present them with MCCV and Y-scrambling together as complementary assessments of model performance. Compared with the literature, Kos et al. [56] classified aflatoxin B_1_ in peanuts at the 8 μg/kg regulatory limit using FTIR spectroscopy, achieving an accuracy of 77.20%. The threshold adopted in the present study (4.0 ppb) is lower than theirs, while the hold-out validation accuracy obtained here (87.50%) is slightly higher than their reported value. Although direct comparisons across different matrices and instruments should be interpreted with caution, these results suggest that the present model shows potential for grading applications at low contamination levels. The discriminating power plot of the SIMCA model (Figure 6d) showed three prominent peaks (around 2150, 1550, and 1400 nm) with high discriminatory power across the spectral range, aligning well with the PCA loadings. Specifically, the region around 1400 nm (corresponding to the vibrations of C-H and O-H bonds in proteins), appeared consistently in both PCA and SIMCA. This observation is consistent with the hypothesis that protein-related constituents may be linked to isolate-specific metabolic differences associated with infection, and a causal link to toxin production pathways remains to be tested. Given that the AFB_1_ concentration in this experiment is at low levels, only subtle biochemical changes occurred in the rice matrix, which can be easily masked by the intrinsic spectral variability of rice. Consequently, overall spectral similarity between the high-AFB_1_ and low-AFB_1_ groups remained high. Nevertheless, these findings suggest that SIMCA provides a feasible approach for the early, rapid screening of AFB_1_-contaminated agricultural products. 

Several non-destructive technologies have been explored for mycotoxin detection, and a comparison with these approaches highlights the practical advantages of the proposed handheld NIR platform. Hyperspectral imaging (HSI) has achieved high accuracy (>96.00%) in differentiating aflatoxigenic fungi [20,57]. However, such systems are typically bulky, costly, and computationally intensive, which restricts their deployment in the field. Laser-induced fluorescence (LIF) has reached accuracies of up to 97.00% in classifying infected mold type, mold infection level, and AFB_1_ concentration, but this performance is obtained on ground rice flour rather than intact grains, and its signals are highly susceptible to light scattering from intact grain surfaces, often necessitating destructive sample grinding for optimal results [58]. Portable mid-infrared (MIR) spectroscopy with ATR sampling requires firm and flat contact between the sample and the crystal, which is difficult to achieve with whole, irregularly shaped cereal grains [17]. In contrast, the handheld NIR sensor used in this study requires no chemical extraction or destructive sample preparation and completes a measurement in approximately 10 s, offering a combination of speed, portability, and accuracy on intact grains that is well suited to rapid, on-site preliminary screening in the food supply chain. The proposed method discriminated rice infected by the aflatoxigenic isolate from rice infected by the non-aflatoxigenic isolate under controlled laboratory conditions, and provided an exploratory grading of early-stage, low-concentration AFB_1_ levels (2.70–23.59 ppb) directly on rice samples, retaining 87.50% overall accuracy on an independent hold-out validation set without destructive grinding or complex optical setups. We acknowledge that this single-point measurement lacks the spatial resolution of HSI for mapping localized contamination; nevertheless, its speed, portability, and demonstrated accuracy on intact grains make it a viable and cost-effective tool for preliminary screening.

However, several limitations of the current study should be acknowledged. One primary concern is that the models were developed with single strains under controlled laboratory conditions, and they may not fully capture the complexity of mixed fungal infections typically encountered in the field. Artificial inoculation likewise cannot fully replicate the heterogeneous nature of natural contamination in real supply chains, in which grain variety, storage history, and regional fungal populations vary simultaneously. More specifically, because only one isolate was used per class, the observed spectral differences between the two infected groups could equally reflect strain-specific characteristics, including growth rate, biomass accumulation, enzyme profile, and substrate preference, rather than toxigenicity itself, and the present design cannot separate these explanations. In addition, fungal biomass was not quantified in this study; consequently, part of the spectral separation between the two infected groups may simply reflect differences in the extent of fungal growth rather than isolate-specific metabolism. Another limitation is that the classification models were trained on a relatively modest sample size, particularly the AFB_1_ grading model built from a single rice matrix and a single toxigenic strain; their generalizability to other rice varieties, origins, and harvest years therefore remains to be verified. In addition, because recovery experiments were not conducted at the 4.0 ppb class boundary itself, the quantitative accuracy in the immediate vicinity of this boundary was not independently verified; class assignments for samples whose concentrations lie close to the boundary should therefore be interpreted as indicative rather than definitive. Furthermore, the relatively small analytical portion (approximately 2–3 g) used for uHPLC-MS/MS analysis represents a limitation, as aflatoxin contamination can be highly heterogeneous and subsampling variability may contribute to the overall analytical uncertainty. Lastly, this study targeted the early, low-concentration contamination window (2.70–23.59 ppb); model performance at higher contamination levels, although expected to benefit from stronger biochemical signatures, was not experimentally evaluated. 

Future work could address these limitations. For example, the sample repository could be expanded to include more rice varieties, naturally contaminated grains, and mixed or multi-strain infections, so that the models can be validated under realistic field conditions. In particular, the models will be validated with a broader panel of toxigenic and non-toxigenic *A. flavus* isolates to test whether the observed spectral differences generalize beyond the two investigated isolates, and direct fungal biomass quantification (e.g., qPCR, ergosterol content, or plate counts) will be incorporated to separate the contribution of fungal growth extent from isolate-specific metabolic differences. In addition, recovery experiments will be performed at the 4.0 ppb class boundary to independently verify the quantitative accuracy in the immediate vicinity of the decision threshold, thereby strengthening the reliability of class assignments for samples close to this boundary. To reduce moisture interference, future work will evaluate naturally stored rice across a broader range of moisture contents, optimize spectral preprocessing and moisture-correction strategies, and determine whether accurate direct measurement can be achieved without a drying step; calibration transfer protocols will also be established for model sharing across instruments and batches. Beyond AFB_1_ content classification, quantitative regression models (e.g., PLSR) will be developed to estimate AFB_1_ concentrations continuously over a wider concentration range, and multi-point scanning or imaging modules may be coupled with the handheld sensor to overcome the lack of spatial resolution and localize contamination hotspots. The validated workflow could ultimately be evaluated in real-world settings such as grain depots and processing lines, moving toward routine on-site screening in the food supply chain.

## 4. Conclusions

This study demonstrated that handheld NIR spectroscopy combined with pattern recognition enables rapid (~10 s), non-destructive discrimination of *A. flavus* inoculation groups in rice. The uHPLC-MS/MS results showed that AFB_1_ levels in the aflatoxigenic *A. flavus*-inoculated samples ranged from 2.70 to 23.59 ppb, on the basis of which the samples were preliminarily separated into low-AFB_1_ (<4.0 ppb) and high-AFB_1_ (≥4.0 ppb) groups for subsequent spectroscopic grading. Pattern recognition (PCA, SIMCA, and OPLS-DA) successfully distinguished the aflatoxigenic *A. flavus*-inoculated, non-aflatoxigenic *A. flavus*-inoculated, and untreated control samples. SIMCA further showed preliminary feasibility for grading AFB_1_ content. The statistical performance of the SIMCA and OPLS-DA algorithms in the discrimination task was acceptable and consistent across the internal and hold-out evaluations; however, given the limited sample size and the low contamination levels investigated, it should be regarded as a preliminary exploration of concept. Validation with a broader panel of aflatoxigenic and non-aflatoxigenic *A. flavus* isolates, together with fungal biomass quantification, is required before practical application. The proposed approach is expected to contribute to regulatory compliance in food safety, reduce costs associated with agricultural product quality control, and mitigate public health risks related to aflatoxin exposure.

## Figures and Tables

**Table 1:** Reference result of *A. flavus*-infected rice samples with aflatoxins in a range of 2.70–23.59 ppb, determined by uHPLC-MS/MS.

Inoculated Strain	Aflatoxin	Minimum	Maximum	Average	SD ^a^
Aflatoxigenic *A. flavus* (CGMCC 3.6304)	AFB_1_	2.70 ppb	23.59 ppb	4.70 ppb	3.48 ppb

^a^ Standard Deviation.

**Table 2:** Intergroup distances among aflatoxigenic *A. flavus*, non-aflatoxigenic *A. flavus*, and control groups, representing the statistical distance between different categories of SIMCA in the principal component space after dimensionality reduction.

	Aflatoxigenic *A. flavus*	Non-Aflatoxigenic *A. flavus*	Control
Aflatoxigenic *A. flavus*	0.00	4.04	5.23
Non-aflatoxigenic *A. flavus*	4.04	0.00	9.98
Control	5.23	9.98	0.00

**Table 3:** Performance comparison of SIMCA and OPLS-DA models in internal and hold-out validations based on accuracy, sensitivity, and specificity.

Model Category	Verification Type	Accuracy (%)	Sensitivity (%)	Specificity (%)
SIMCA	Internal validation	100.00	100.00	100.00
	Hold-out validation	96.67	96.67	100.00
OPLS-DA	Internal validation	95.90	95.90	99.18
	Hold-out validation	96.67	96.67	98.33

**Table 4:** Variance and root mean square error of cross-validation (RMSECV) for each factor in the OPLS-DA model across aflatoxigenic *A. flavus*, non-aflatoxigenic *A. flavus*, and control groups.

	Aflatoxigenic *A. flavus*		Non-Aflatoxigenic *A. flavus*		Control	
	Cumulative	RMSECV	Cumulative	RMSECV	Cumulative	RMSECV
Factor 1	30.87	0.27	40.97	0.30	47.04	0.21
Factor 2	53.95	0.18	66.33	0.23	77.04	0.20
Factor 3	93.85	0.12	93.78	0.19	78.58	0.21
Factor 4	97.06	0.11	96.80	0.20	97.09	0.21
Factor 5	98.09	0.12	97.52	0.20	98.35	0.21

**Table 5:** Internal and hold-out validation results of the SIMCA model for AFB_1_ content classification.

Model Category	Verification Type	Accuracy (%)	Sensitivity (%)	Specificity (%)
SIMCA	Internal validation	100.00	100.00	100.00
	Hold-out validation	87.50	100.00	66.67

## Data Availability

The original data presented in the study are included in the article and Supplementary Material. Further inquiries can be directed to the corresponding author.

## Supplementary Materials

The following supporting information can be downloaded at https://www.mdpi.com/article/10.3390/foods15203762/s1: Table S1: Pooled confusion matrix of the OPLS-DA model over 200 random 4:1 partitions; Table S2: Pooled confusion matrix of the SIMCA model over 200 random 4:1 partitions; Table S3: Performance of the SIMCA and OPLS-DA models evaluated by Monte Carlo cross-validation; Table S4: Summary of the Y-scrambling test for the OPLS-DA model; Table S5: uHPLC-MS/MS gradient elution program for aflatoxin analysis; Table S6: MCCV and Y-scrambling test results of the AFB_1_ grading model for each number of components (ncomp = 1–10); Table S7: Summary of the Y-scrambling test for the SIMCA model; Figure S1: Test-set accuracy distributions of the SIMCA and OPLS-DA models; Figure S2: Y-scrambling permutation test for the OPLS-DA model; Figure S3: Representative images at 24, 48 and 72 h post-inoculation; Figure S4: Y-scrambling permutation test for the SIMCA model.
