# Supplementary material for: Handheld Near-Infrared Spectroscopy Integrated with Pattern Recognition for Non-Destructive Discrimination of Rice Infected by an Aflatoxigenic Versus a Non-Aflatoxigenic Aspergillus flavus Isolate
Source: Foods. 2026 Oct 21;15(20):3762. doi: 10.3390/foods15203762 (PMC13704131; doi:10.3390/foods15203762)
Supplement: Supplementary file 1 [file foods-15-03762-s001.zip › foods-4572685-supplementary.pdf]

Table S1. Pooled confusion matrix of the OPLS-DA model over the 200 random 4:1 partitions (6400 test-set predictions in total: 2200 control, 2000 aflatoxigenic, and 2200 non-aflatoxigenic instances). Rows indicate the actual class and columns indicate the predicted class; diagonal entries are correct classifications. Notably, no aflatoxigenic instance was misclassified as control (0/2000), and the pooled accuracy across all partitions was 95.64% (6121/6400)

	Control	Aflatoxigenic	Non-aflatoxigenic
Control	2055	51	94
Aflatoxigenic	0	1967	33
Non-aflatoxigenic	98	3	2099

Table S2. Pooled confusion matrix of the SIMCA model over the 200 random 4:1 partitions (6400 test-set predictions in total: 2200 control, 2000 aflatoxigenic, and 2200 non-aflatoxigenic instances). Rows indicate the actual class and columns indicate the predicted class; diagonal entries are correct classifications. The pooled accuracy (6025/6400 = 94.14%) is consistent with the mean accuracy reported in **Table S3.**

	Control	Aflatoxigenic	Non-aflatoxigenic
Control	1843	257	100
Aflatoxigenic	0	1997	3
Non-aflatoxigenic	1	14	2185

Table S3. Performance of the **SIMCA and OPLS-DA** models evaluated by Monte Carlo cross-validation (200 random stratified 4:1 training/validation partitions of the full dataset, n = 154). Values are presented as mean ± standard deviation, with 95% confidence intervals obtained from the 2.5th and 97.5th percentiles of the respective distributions. Sensitivity and specificity are macro-averaged over the three classes (control, aflatoxigenic *A. flavus*, and non-aflatoxigenic *A. flavus*).

Model	Metric	Mean_SD	CI_95
SIMCA	Accuracy	94.14 ± 4.53	84.4-100.0
SIMCA	Sensitivity	94.31 ± 4.40	84.8-100.0
SIMCA	Specificity	97.12 ± 2.22	92.3-100.0
OPLS-DA	Accuracy	95.64 ± 3.52	87.5-100.0
OPLS-DA	Sensitivity	95.72 ± 3.44	87.9-100.0
OPLS-DA	Specificity	97.81 ± 1.77	93.7-100.0

Table S4. Summary of the Y-scrambling test (200 permutations) for the **OPLS-DA** model. The true model and all permuted models share the identical preprocessing pipeline and number of latent variables, with performance assessed by LOOCV. The p value was calculated as (1 + number of permuted models achieving an accuracy equal to or higher than the true model) / (200 + 1).

Item	Value
Permutations	200
Permuted mean (%)	32.69
Permuted SD (%)	5.42
Permuted max (%)	50
p value	$p < 0.005$

Table S5. uHPLC-MS/MS gradient elution program for aflatoxin analysis (Mobile Phase A: 0.1% formic acid in water; Mobile Phase B: 0.1% formic acid in acetonitrile).

Time (min)	Mobile Phase A (%)	Mobile Phase B (%)
0.00	95	5
2.00	95	5
2.10	60	40
7.00	45	55
10.00	10	90
10.50	95	5
13.00	95	5
15.00	95	5

Table S6. Monte Carlo cross-validation (MCCV) and Y-scrambling test results of the AFB_1 grading model for each number of components (ncomp = 1–10)

ncomp	acc_mean	acc_sd	bal_mean	bal_sd	perm_p
1	41.40 ± 14.70	14.70	40.2	15.2	0.697
2	51.70 ± 12.30	12.30	44.5	12.3	0.612
3	60.20 ± 15.20	15.20	59.5	15.1	0.219
4	63.10 ± 16.20	16.20	62.8	16.4	0.154
5	63.10 ± 15.70	15.70	57.7	16.5	0.100
6	61.80 ± 10.00	10.00	52.5	11.1	0.100
7	61.70 ± 7.10	7.10	50.6	7.6	0.080
8	62.30 ± 4.00	4.00	50.10	4.0	0.035
9	62.50 ± 0.00	0	50.00	0	1.000
10	62.50± 0.00	0	50.00	0	1.000

Note: ncomp, number of principal components; MCCV accuracy, mean accuracy across 200 Monte Carlo cross-validation partitions; MCCV balanced accuracy, mean balanced accuracy across the same 200 partitions; Y-scrambling p-value, empirical p-value calculated as $p = (1 + k) / (N + 1)$, where k is the number of permuted models achieving accuracy equal to or higher than the original (unpermuted) model, and N is the total number of permutations (200). In each MCCV partition, the development set was split into training and test sets at a 4:1 ratio under class stratification, and the same 200 partitions were used for all ncomp values. Preprocessing was identical to that used for the final AFB_1 grading model. The Y-scrambling test was performed for each ncomp value by randomly permuting the class labels while keeping the spectral data unchanged.

Table S7. Summary of the Y-scrambling test (200 permutations) for the SIMCA model. The true model and all permuted models share the identical preprocessing pipeline and number of latent variables, with performance assessed by LOOCV. The p value was calculated as (1 + number of permuted models achieving an accuracy equal to or higher than the true model) / (200 + 1).

Item	Value
Permutations	200
Permuted mean (%)	32.79
Permuted SD (%)	4.18
Permuted max (%)	44.16
p value	$p < 0.005$

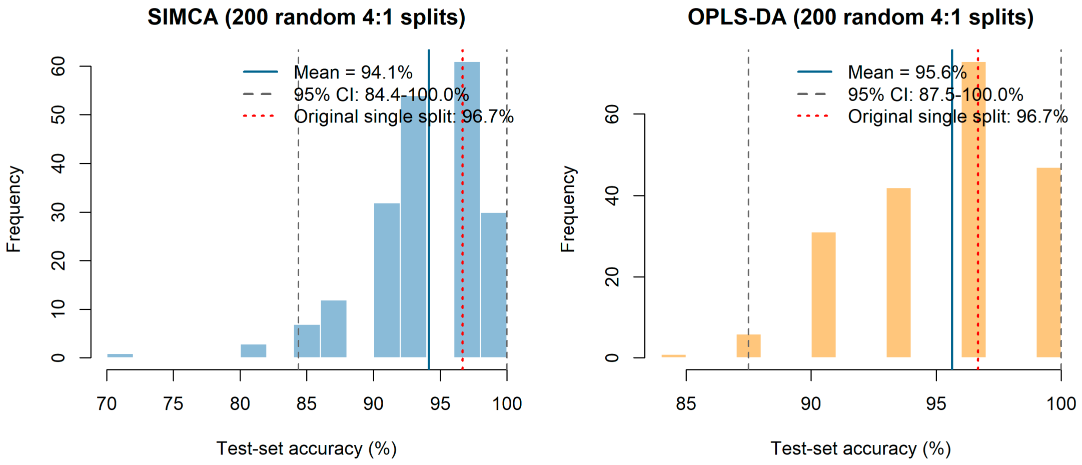

Figure. S1. Test-set accuracy distributions of the SIMCA (left) and OPLS-DA (right) models over 200 random stratified 4:1 partition. Solid line: mean; dashed lines: 95% percentile interval; red dotted line: accuracy of the original single partition (96.67%).

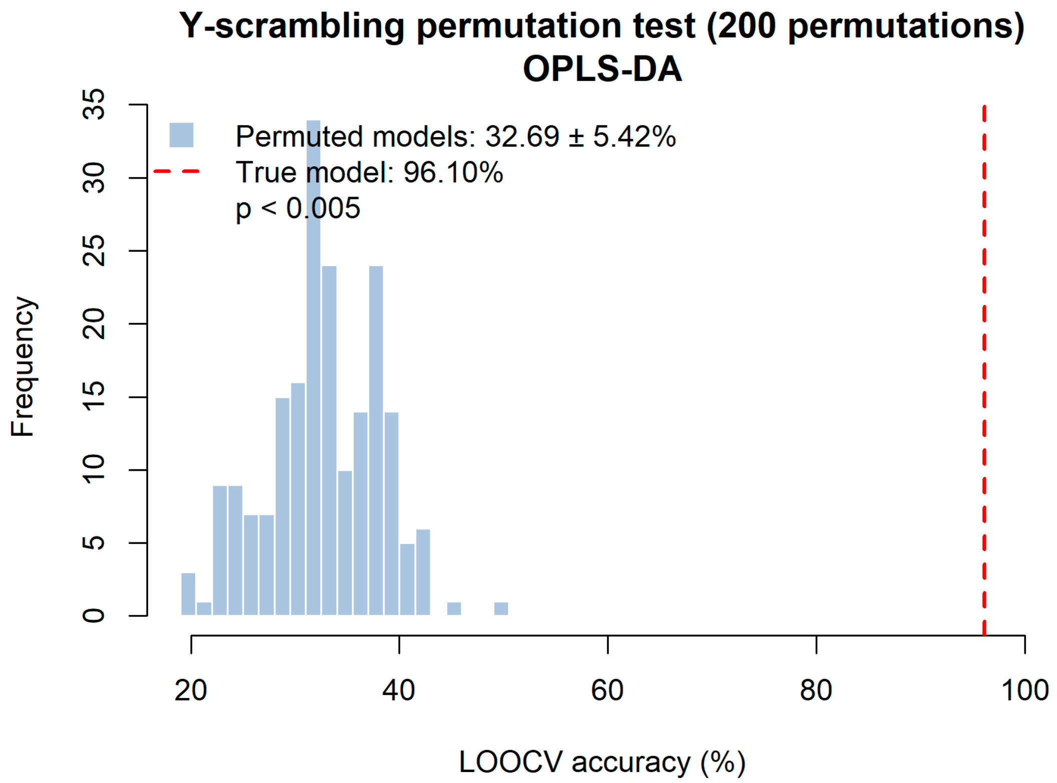

Figure S2. Y-scrambling permutation test (200 permutations) for the OPLS-DA model. Class labels were randomly permuted while keeping the spectral matrix unchanged; for each permutation, a model was rebuilt using the identical preprocessing pipeline and the same number of latent variables, and its performance was assessed by leave-one-out cross-validation (LOOCV). Histogram: distribution of LOOCV accuracies of the 200 permuted models (mean ± SD: 32.69 ± 5.42%; maximum: 50.00%), centered at the theoretical chance level (33.3%). No permuted model approached the true model's performance ($p < 0.005$), confirming that the classification is driven by genuine spectral–class relationships rather than chance correlations.

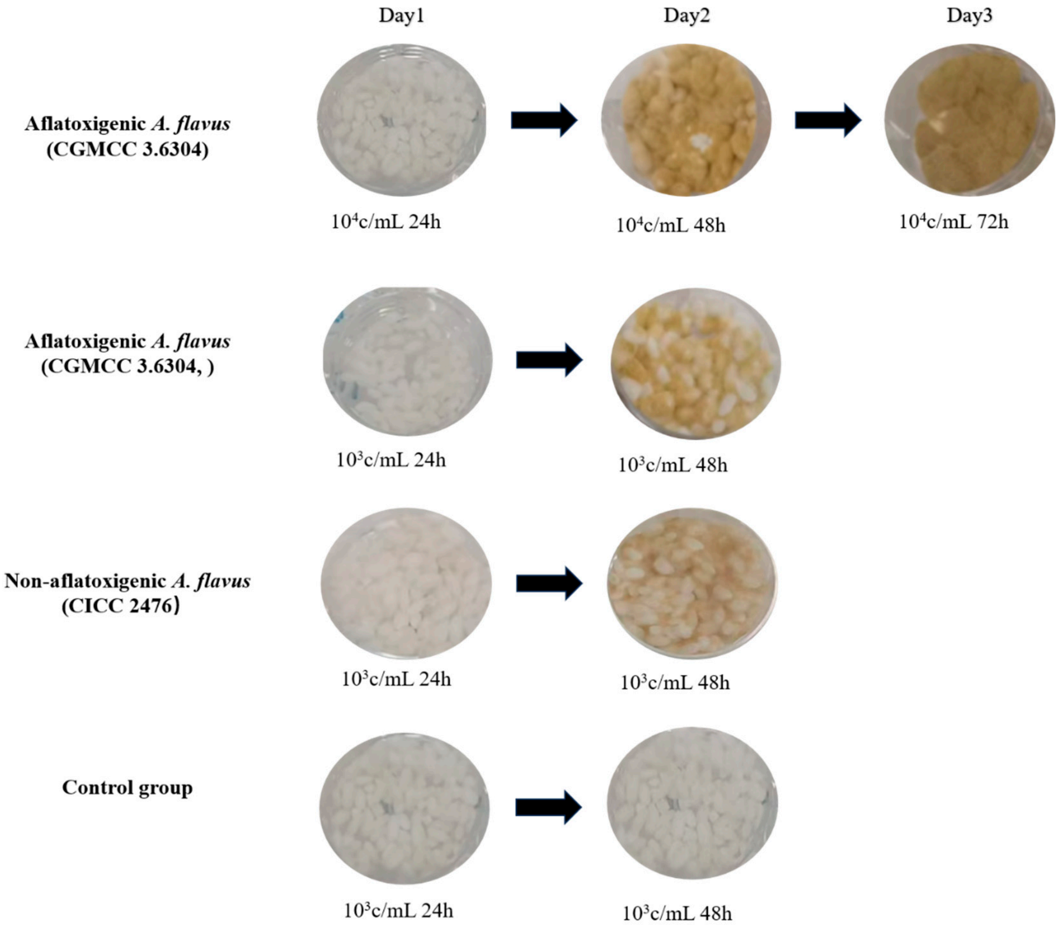

Figure S3. Representative images at 24 and 48 h post-inoculation for all conditions, and at 72 h for the 10^4 CFU/mL aflatoxigenic condition.

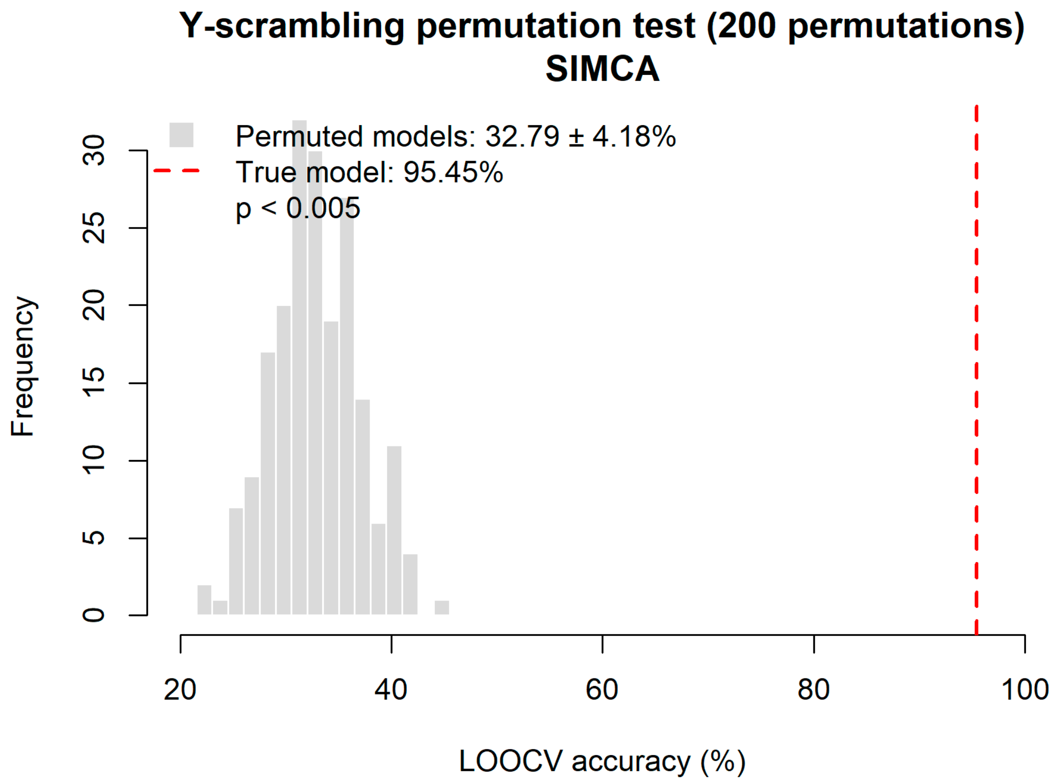

Figure S4. Y-scrambling permutation test (200 permutations) for the SIMCA model. Class labels were randomly permuted while keeping the spectral matrix unchanged; for each permutation, the three class models were rebuilt using the identical preprocessing pipeline, and performance was assessed by leave-one-out cross-validation (LOOCV) with nearest-class assignment based on normalized Q residuals. Histogram: distribution of LOOCV accuracies of the 200 permuted models (mean ± SD: 32.79 ± 4.18%; maximum: 44.16%), centered at the theoretical chance level (33.3%). No permuted model approached the true model's performance ($p < 0.005$), confirming that the classification is driven by genuine spectral–class relationships rather than chance correlations.
